# Does Long-Term Sport Practice Facilitate the Development of Idiopathic Bradycardia Requiring Early Pacemaker Implantation During the Course of Life?

**DOI:** 10.3390/jcdd12030102

**Published:** 2025-03-15

**Authors:** Sergei Bondarev, Leonardo Brotto, Francesca Graziano, Alberto Cipriani, Domenico Corrado, Alessandro Zorzi

**Affiliations:** Department of Cardiac, Thoracic and Vascular Sciences and Public Health, University of Padua, 35122 Padova, Italy; sergei.bondarev@unipd.it (S.B.); francesca.graziano@unipd.it (F.G.); alberto.cipriani@unipd.it (A.C.); domenico.corrado@unipd.it (D.C.)

**Keywords:** atrioventricular block, athletes, epigenetic, sinus bradycardia, sports cardiology

## Abstract

*Background*: Sinus bradycardia and first-/second-degree atrioventricular (AV) block in athletes are traditionally considered secondary to increased vagal tone and therefore reversible. However, recent studies have suggested that they may persist even after the cessation of physical activity, and combined with the effects of aging, lead to the earlier onset of clinically significant bradyarrhythmias. *Methods:* We evaluated the correlation between lifetime sport practice and the age of the onset of premature (≤70 years old) idiopathic sinoatrial node or AV node dysfunction requiring pacemaker (PM) implantation. *Results*: Of the 1316 patients followed up with at our PM clinic in 2024, we included 79 (6%) who received a PM when they were ≤70 years old for bradyarrhythmias in the absence of secondary causes. Nineteen (24%) had engaged in at least 6 h of sports/week for ≥20 years and were classified as former athletes. For comparison, former athletes who received a PM for idiopathic bradycardia at >70 years old were 6% (*p* < 0.001). In the group ≤70 years old, the average age of PM implantation was 62.8 years in non-athletes versus 57.9 years in former athletes (*p* = 0.03). The main reason for PM implantation was AV block in both subgroups. Among former athletes, the correlation between the lifetime volume of sports activity and the age of PM implantation reached borderline statistical significance (*p* = 0.08). Echocardiography at the time of implant did not reveal significant differences between former athletes and non-athletes. *Conclusions:* In a cohort of patients who received a PM for bradyarrhythmia before the age of 70 years old in the absence of secondary causes, former athletes were implanted on average ≈5 years before non-athletes. This may suggest a contributing role of cumulative sports activity volume in the development of idiopathic sinus/AV node dysfunction.

## 1. Introduction

Intense training modulates the autonomic nervous system, increasing vagal tone at rest and sympathetic activity during exertion [1]. According to the classical theory, sinus bradycardia and first- and second-degree Mobitz I (supra-Hisian) atrioventricular (AV) blocks are considered secondary to the high vagal tone, and as such, they should be completely reversible upon cessation of physical activity [2,3]. Indeed, according to international and Italian guidelines for granting eligibility for competitive sports, even extreme sinus bradycardias (up to 30 beats per minute), first-degree AV block and second-degree Mobitz type I AV block are considered physiological in athletes who engage in high-volume endurance sports, provided there is a complete normalization of heart rate and AV conduction during exertion [4,5,6]. However, it is a common clinical experience that some individuals with a long history of sports activity remain bradycardic even after ceasing training, contradicting the theory that athlete’s bradycardia is simply a result of reversible hypervagotonia [7]. There are two possible mechanisms that could explain persistent bradycardia in athletes: stress damage (oxidative, increased activity of the renin-angiotensin system, etc.) that could eventually lead to the death of cells in the sinoatrial node (SN) or atrioventricular node (AVN) and their replacement with fibrous tissue [8,9,10,11,12], and the down-regulation of ion channel gene transcription, which plays a significant role in regulating the activity of the AVN and SN [13,14,15,16]. Nevertheless, the vast majority of people who engage in intense physical activity do not exhibit any rhythm disturbances that would require pacemaker (PM) implantation after ending their career [17]. It is therefore reasonable to assume that “damage” from sports is not sufficient to cause symptomatic bradycardia, but it could contribute to the age-dependent pathological process linked to the development of idiopathic SN or AVN dysfunction in adults and the elderly. In other words, the hypothesis is that a degenerative pathology related to fibrosis of the SN or AVN due to aging (Lev–Lenègre disease) might be facilitated and thus manifest earlier in patients who have engaged in high-volume training.

We sought to evaluate the prevalence of a history of long-lasting sports activity in a cohort of individuals who received a PM relatively early during their life course (≤70 years old) for idiopathic SN or AVN dysfunction and whether age at the time of implant was influenced by previous athletic career and cumulative training volume.

## 2. Materials and Methods

This is a single-center, cross-sectional observational study conducted among patients evaluated over a 1-year period at the PM control clinic of the Cardiology Department at the Padova University Hospital. The study was approved by the Ethical Committee of Padua (Code 5763/AO/23). Data supporting the study are available from the corresponding author upon reasonable request.

Patients were included in the study if they fulfilled the following criteria:

- PM implanted for premature (<70 years old) brady-arrhythmia. This arbitrary age-cut off was used taking into account the results of international registries showing that most PM implantations occur later [18,19];
- Idiopathic dysfunction of the SN or AVN, thus excluding individuals with bradycardia secondary to other cardiac diseases (ischemic, valvular, or post-surgical). Patients who required PM implantation for vasovagal syncope were also excluded;
- No previous interventional cardiology procedures;
- Regular clinical–instrumental follow-up;
- Ability to provide consent (in cases of doubt, a mini-mental state examination with a cut-off score ≥ 24 was used).

The following variables were collected for each patient:

- Personal and family history;
- Symptoms and ECG characteristics before PM implantation;
- Type of PM and programming mode;
- Echocardiography performed prior to PM implantation;
- History of sports activity:
  ○ Type of sport(s) practiced, classified according to the European Society of Cardiology in four categories: group A (precision sports); group B (strength sports); group C (mixed sports); and group D (endurance sports);
  ○ Number of years of sport practice (excluding group A disciplines because of their low cardiovascular demands);
  ○ Mean number of weekly training hours per year of sport. The total volume of sport activity was then estimated by multiplying the mean number of training hours per week * years of sport activity.

Patients were arbitrarily classified as former athletes if for at least 20 years of their lives, they had been engaged in at least 6 h of physical activity per week excluding group A disciplines. All other respondents were classified as non-athletes.

### Statistical Analysis

Qualitative variables were expressed as number (%), quantitative variables with a normal distribution as mean (standard deviation), and those with a non-normal distribution as median (25th and 75th percentile values). The Shapiro–Wilk test was used to evaluate the normality of qualitative variables. For the comparison of variables with a normal distribution, Student’s *t*-test was used, while the Mann–Whitney U test was used for non-normal variables. For comparisons between qualitative variables, the chi-square test or Fisher’s exact test was used as appropriate. In the subgroup of athletes, the correlation between age at PM implantation and training volume was assessed with a univariate linear regression analysis. A *p*-value < 0.05 was considered significant. The database was created using RedCap hosted at the University of Padua. The statistical analysis was performed using SPSS ver. 28 (IBM, Armonk, NY, USA).

## 3. Results

The total population that underwent a follow-up visit at the PM clinic of the University Hospital of Padova during the study period was 1316 patients. Of these, only 79 (6%) met the study inclusion criteria, and 19/79 (24%) were classified as former athletes. For comparison, we evaluated previous sport activity in 214 patients with idiopathic bradycardia who received a PM after the age of 70 years old (on average 79 years old) and without cognitive impairment: only 12 (6%) were classified as former athletes according to our definition, a significantly lower proportion than that of patients who received a PM before 70 years old (<0.001).

### 3.1. Former Athletes’ Characteristics

Among the athletes, the average age of starting sports activity was 15.3 ± 7.26 years, with a median of 15 (range 10–17) years. On average, they had participated in sports activities for 45 ± 10 years, with a median of 45 (range 39–52) years. The average number of hours per week they engaged in sports was 9.5 ± 5.2. Regarding sport disciplines, 3 practiced mainly strength sports, 12 mixed sports, and 14 endurance sports. Four athletes (21%) were cyclists, with a median annual cycling distance of 8000 (range, 4000–20,000 km). Nine (47%) practiced running, six had run at least one marathon, and the others at least one half-marathon.

### 3.2. Comparison Between Athletes and Non-Athletes

**Figure 1:**
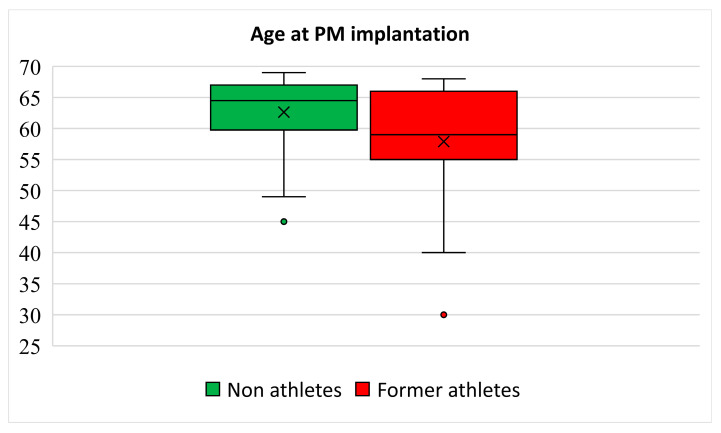
Box-and-whiskers plot showing age at PM implantation in non-athletes and former athletes.

Most non-athletes (41, 68%) and athletes (16, 84%) were male (*p* = 0.18) with a similar age at the time of evaluation. All but two non-athletes and all athletes were Caucasian. A family history of cardiac disease or sudden death was reported by 5 (8%) non-athletes versus 0 athletes (*p* = 0.27). The age at PM implantation was significantly higher in non-athletes compared to that of athletes (median, 64 versus 59 years; *p* = 0.03) (Figure 1).

The symptoms leading to bradycardia diagnosis, ECG characteristics, history of atrial fibrillation, and reasons for PM implantation are reported in Table 1. Apart from a higher incidence of atrial fibrillation history in non-athletes, no other variables showed differences between the two groups.

The pre-implantation echocardiographic parameters are reported in Table 2. No differences between the two groups were observed.

### 3.3. Correlation Between Training Volume and Age at PM Implantation in Athletes

**Figure 2:**
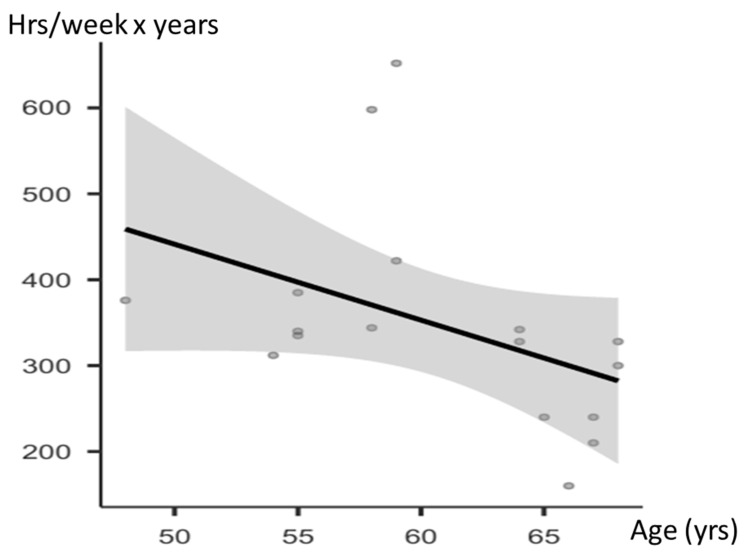
Correlation between age of PM implantation and training volume (mean hours per week × years of training).

Figure 2 shows a linear regression analysis to correlate age at PM implantation and the cumulative volume of physical activity in the athlete subgroup. The correlation showed borderline statistical significance (*p* = 0.08).

## 4. Discussion

This single-center, cross-sectional observational study analyzed 1316 patients from the PM clinic at Padova University Hospital, focusing on 79 individuals who underwent “premature” pacemaker (PM) implantation due to idiopathic sinoatrial node (SN) or atrioventricular node (AVN) dysfunction. We decided to focus on “premature” PM implantation because we hypothesized that the potential deleterious effects of exercise would have been more evident in this age group, compared to elderly patients in which the progressive degeneration of the cardiac conduction system is more likely to prevail. In this subgroup of 79, 19 patients (24%) were identified as former athletes based on their participation in mixed or endurance sports, maintaining a training load of ≥6 h for over 20 years.

The key findings include the following: (1) a higher proportion of former athletes among patients with idiopathic bradycardia received “premature” PM compared to those who received a PM at a later age; (2) athletes under 70 required PM approximately 5 years earlier than non-athletes despite similar initial characteristics and symptoms; (3) a trend toward an inverse correlation between total training load and age at PM implantation among the 19 athletes; (4) echocardiography at the time of implant did not reveal differences between former athletes and non-athletes, showing that exercise-induced remodeling had already subsided while bradycardia persisted.

Training is recognized for increasing parasympathetic activity in athletes, especially those in endurance sports, commonly leading to sinus bradycardia and first-degree AV block, and infrequently, second-degree type 1 AV block [1,2]. However, previous studies have demonstrated that dual autonomic system blockage with atropine and propranolol does not fully restore SAN and AVN functions in athletes, suggesting intrinsic underlying changes [20,21,22].

A possible explanation to this finding derives from recent animal studies indicating that prolonged physical activity may downregulate ion channel gene expression. This implies that detraining may not fully reverse these exercise-induced changes, potentially leading to more frequent and earlier symptomatic sinus bradycardia or AV block in former athletes [13,14,15,16].

Another theory suggests that SN and AVN damage could be linked to the direct harmful effects of catecholamines, peroxides, and severe metabolic acidosis. Elevated catecholamine levels stimulate β-adrenergic receptors in cardiomyocytes, increasing adenylate cyclase activity through Gs protein synthesis. This conversion of ATP to cAMP activates a protein kinase that opens Ca2+ channels, elevating intracellular Ca2+ levels. The activation of Ca2+-dependent proteases and Na+/Ca2+ exchange channels accelerates oxidative processes and the buildup of reactive oxygen species, potentially leading to cardiomyocyte necrosis and apoptosis [8,9,10,11,12].

Whether prolonged and intense training may predispose to persistent SAN and AVN remodeling leading to a greater predisposition towards symptomatic bradycardia during the life course remains debated, but some observational studies seem to support this possibility [17]. Among former Swiss cyclists, Baldesberger et al. observed more frequent SAN dysfunction compared with age-matched controls [23]. In a comprehensive Swedish registry study involving 209,108 endurance-trained former cross-country skiers and 532,290 individuals from the general population, a higher long-term incidence of bradycardia and PM implantations was noted in former skiers compared to non-skiers, especially those with the most races and fastest completion times, a trend not seen in female participants [24]. Our previous study also found that former professional endurance athletes required PM implantation significantly earlier than non-endurance athletes and control groups, suggesting a potential long-term impact of intense sports activities on the cardiac conduction system [25].

The results of the current study support the hypothesis that intense and prolonged sports activity may accelerate the development of progressive SN or AVN dysfunction compared to non-athletes. Animal studies suggest that this mechanism may be reversible targeting its molecular bases [14,26]. However, considering that out of more than 1300 patients with PM followed up with at our hospital, only 19 former athletes required ’premature’ implantation, it appears that the deleterious effects of prolonged sports activity on the cardiac excitation-conduction system become clinically significant only in a minority of cases. Moreover, the 24% proportion of former athletes among those with premature PM implantation could potentially overestimate the risk associated with training, as individuals who lead an active lifestyle are more likely to become symptomatic in the event of bradycardia.

It is noteworthy that in our cohort, the majority of athletes received a PM for AV block, whereas previous studies suggested that prolonged physical exercise impairs SAN rather than AVN function [27]. This discrepancy might reflect a greater propensity for PM implantation in young individuals with second or third-degree AV blocks rather than sinus bradycardia, which is often treated more conservatively. Despite the high proportion of AV block as a reason for PM implantation, only 4 of 19 (20%) former athletes displayed bundle branch block on pre-implantation ECGs, suggesting that the block’s origin was in the AVN rather than the ventricular cardiac conduction system, which is less likely to be affected by prior sporting activity. Another interesting finding was the male predominance of former athletes requiring premature PM implantation, which seems to support the observation of Svedberg et al. [23] that previous sports activity is associated with a higher risk of PM implantation in males but not in females. However, in our study, non-athletes with premature PM implantation also showed a male predominance. Moreover, female sport in Italy was relatively uncommon decades ago.

### Study Limitations

Because of our strict inclusion criteria, the sample size, particularly of former athletes, was relatively small, which might affect the generalizability of the results. Additionally, the retrospective nature of the study and reliance on historical medical records for sports history may introduce recall biases or inaccuracies. Finally, as always, we have to keep in mind that association does not equal a cause–effect relationship, and this is particularly true for retrospective observational studies. Further research with larger, prospectively followed cohorts would be beneficial to confirm these findings and potentially explore the mechanisms underlying these observations.

## 5. Conclusions

In conclusion, our study corroborates the idea that long-term, intense endurance training may predispose former amateur athletes to an earlier development of SN or AVN dysfunction. These findings are consistent with our previous research on former professional athletes [24]. However, it is important to remember that numerous studies have demonstrated that sports activity is generally beneficial and associated with lower mortality rates in the general population. Additionally, only a small minority of former athletes ever requires pacemaker implantation during their lifetime. Other factors, such as age-dependent degeneration or genetic predisposition, are likely necessary for clinically significant bradycardia to manifest in a former athlete.

Nonetheless, these findings challenge the traditional perception of athlete’s heart as merely an adaptive response and underscore the importance of monitoring for potential long-term adverse effects of extreme endurance sports on the cardiac conduction system. Future research should focus on identifying specific training thresholds that contribute to these risks and developing guidelines to mitigate them for athletes engaged in long-term, high-intensity sports.

## Figures and Tables

**Table 1:** Comparison between pre-implantation symptoms, ECG findings, and the diagnosis between athletes and non-athletes.

	Non-Athletes (*n* = 60)	Athletes (*n* = 19)	*p*-Value
**Pre-implantation symptoms:**			
Dizziness, *n* (%)	9 (15.0%)	2 (10.5%)	0.62
Fatigue, *n* (%)	11 (18.3%)	4 (21.1%)	0.79
Exertional dyspnea, *n* (%)	9 (15.0%)	5 (26.3%)	0.26
Chest pain, *n* (%)	3 (5.0%)	1 (5.3%)	0.96
Syncope, *n* (%)	31 (51.7%)	8 (42.1%)	0.47
Asymptomatic, *n* (%)	16 (21.7%)	4 (21.1%)	0.62
**Pre-implantation ECG:**			
Right bundle branch block, *n* (%)	8 (13.3%)	2 (10.5%)	0.75
Left bundle branch block, *n* (%)	2 (3.3%)	2 (10.5%)	0.21
Atrial fibrillation, *n* (%)	11 (18.3%)	0 (0%)	0.04
**Pre-implantation diagnosis:**			
Sick sinus syndrome, *n* (%)	15 (25.0%)	3 (15.8%)	0.40
Second-degree Mobitz I AV block, *n* (%)	2 (3.3%)	0 (0%)	0.42
Second-degree Mobitz II AV block, *n* (%)	20 (33.3%)	8 (42.1%)	0.49
Third-degree AV block, *n* (%)	26 (43.3%)	9 (47.4%)	0.76
**Pacemaker pacing mode:**			
AAI (with back-up DDD), *n* (%)	15 (25.0%)	3 (15.8%)	0.54
VVI, *n* (%)	9 (15.0%)	0	0.11
DDD, *n* (%)	36 (60.0%)	16 (84.2%)	0.06

**Table 2:** Pre-implantation echocardiographic findings.

	Non-Athletes (*n* = 60)	Athletes (*n* = 19)	*p*
Left atrial volume, mean (SD)	34.2 (10.3)	32.2 (5.4)	0.62
Left atrial systolic area, mean (SD)	21.8 (5.2)	20.7 (2.7)	0.63
Right atrial volume, mean (SD)	27.1 (12.5)	29.8 (9.2)	0.65
Right atrial systolic area, mean (SD)	20.1 (6.5)	18.7 (1.8)	0.65
Left ventricular ejection fraction, mean (SD)	58.7 (7.9)	62.3 (8.3)	0.18
Left ventricular diastolic volume, mean (SD)	57.5 (11.2)	62.3 (12.8)	0.24
Left ventricular systolic volume, mean (SD)	24.8 (7.9)	22.8 (8.5)	0.50
Interventricular septal thickness, mean (SD)	11.6 (2.3)	12.1 (4.1)	0.64
Left ventricular end-diastolic diameter, mean (SD)	49.6 (5.7)	47.9 (6.1)	0.47
Posterior wall thickness, mean (SD)	10.7 (1.6)	11.2 (2.2)	0.41
Right ventricular shortening fraction, mean (SD)	44.4 (8.3)	44.4 (4.8)	0.99
Right ventricular end-diastolic area, mean (SD)	20.6 (5.4)	23.3 (4.2)	0.31
Right ventricular end-systolic area, mean (SD)	13.6 (6.0)	14.0 (5.1)	0.87
Tricuspid Annular Plane Systolic Excursion (TAPSE), mean (SD)	24.1 (5.2)	24.0 (7.7)	0.97
Pulmonary artery pressure, mean (SD)	26.5 (9.3)	21.3 (9.6)	0.22
Inferior vena cava collapsibility index, mean (SD)	59.4 (11.1)	59.4 (7.3)	0.99

## Data Availability

The data supporting this study are available from the corresponding author upon reasonable request.

## Abbreviations

The following abbreviations are used in this manuscript:

AV	Atrioventricular
AVN	Atrioventricular node
PM	Pacemaker
SN	Sinus node
